# Vitamin D receptor gene polymorphisms modulate the clinico-radiological response to vitamin D supplementation in knee osteoarthritis

**DOI:** 10.1186/1755-8166-7-S1-P13

**Published:** 2014-01-21

**Authors:** Divya Sanghi, RN Srivastava, Sarita Agarwal

**Affiliations:** 1 King George’s Medical University, Near Daligunj Chauraha, Lucknow, India; 2 Post Graduate Institute of Medical sciences, Raibareily Road, Lucknow, India

## Background

Vitamin D receptor (VDR) gene plays an important role in bone mass regulation and is demonstrated in human articular chondrocytes of cartilage. We have previously shown a beneficial effect of vitamin D on symptomatic improvement in Osteoarthritis knee (KOA) patients. This study investigated whether the clinic-radiological response to vitamin D was modulated by VDR gene.

## Materials and methods

This randomized placebo-controlled trial recruited 103 KOA cases as per American College of Rheumatology (ACR) guideline having vitamin D insufficiency (25(OH)D≤50 nmol/L). Enrolled cases were randomly allocated in two groups to receive placebo (51) and vitamin D (52). Primary outcome measures: pain and functional disability which were recorded by knee specific WOMAC index and secondary outcome measure were radiological features (joint space width and osteophytes). The serum levels of vitamin D were assessed by ELISA method using IDS, UK kit. Detection of VDR polymorphisms (Taq1 & Apa I) were done by PCR-RFLP technique. 25(OH)D levels, clinical and radiological features were recorded at baseline and at one year follow up.

## Results

At one year, in vitamin D group, TT genotype of TaqI polymorphism showed increment in 25(OH)D levels in comparison to Tt and tt genotype whereas in placebo group it remained same. No such association was observed for ApaI polymorphism. In clinical features, pain significantly increased in Tt and tt genotype of placebo group whereas it decreased, although not significant in each genotype of vitamin D group. Total WOMAC scores significantly decreased in both Tt and TT(p<0.05) genotypes in vitamin D group, whereas functional disability only in Tt(p<0.05). In radiological features, decreased Medial-JSW was observed in tt genotype and increased Osteophyte was observed in TT and Tt genotype of placebo group whereas no significant changes were noted in vitamin D group. No such effect was observed with ApaI polymorphism.

## Conclusions

VDR gene polymorphisms influence the clinico-radiological response to vitamin D supplementation in KOA Bottom of Fo with low 25-OHD levels.

